# Feasibility of a Combined Mobile-Health Electrocardiographic and Rapid Diagnostic Test Screening for Chagas-Related Cardiac Alterations

**DOI:** 10.3390/microorganisms9091889

**Published:** 2021-09-06

**Authors:** Michele Spinicci, Carlo Fumagalli, Niccolò Maurizi, Enrico Guglielmi, Mimmo Roselli, Herlan Gamboa, Marianne Strohmeyer, Veronica Poma, Roberto Vargas, Iacopo Olivotto, Alessandro Bartoloni

**Affiliations:** 1 Department of Experimental and Clinical Medicine, University of Florence, 50134 Florence, Italy; michele.spinicci@unifi.it (M.S.); carlo.fumagalli@unifi.it (C.F.); guglielmi75@yahoo.it (E.G.); mimrose@hotmail.com (M.R.); marianne.strohmeyer@unifi.it (M.S.); 2 Infectious and Tropical Diseases Unit, Careggi University Hospital, 50134 Florence, Italy; 3 Cardiomyopathy Unit, Cardiothoracic and Vascular Department, Careggi University Hospital, 50134 Florence, Italy; niccolo.maurizi@gmail.com; 4 Cardiology Service, University Hospital of Lausanne, CH-1011 Lausanne, Switzerland; 5 Facultad Integral del Chaco, Universidad Autónoma Gabriel René Moreno, Camiri, Bolivia; herlan.gamboa@hotmail.com; 6 Escuela de Salud del Chaco Tekove Katu, Gutierrez, Bolivia; kollita2009@hotmail.com; 7 Programa Nacional de Chagas, Ministerio de Salud, La Paz, Bolivia; sedessantacruz@hotmail.com

**Keywords:** Chagas disease, Chagas Stat-Pak, chronic Chagas cardiomyopathy, mHealth, telemedicine, seroprevalence, Bolivia, Chaco

## Abstract

Background: Chronic Chagas cardiomyopathy (CChC) is the most common cause of death related to Chagas disease (CD). The aim of this study was to assess the feasibility of a combined rapid diagnostic test (RDT) and electrocardiographic (ECG) screening in a remote rural village of the Bolivian Chaco, with a high prevalence of CChC. Methods: Consecutive healthy volunteers > 15 years were enrolled in the community of Palmarito (municipality of Gutierrez, Santa Cruz Department, Bolivia) in February 2019. All patients performed an RDT with Chagas Stat-Pak^®^ (CSP, Chembio Diagnostic System, Medford, NY, USA) and an ECG by D-Heart^®^ technology, a low-cost, user-friendly smartphone-based 8-lead Bluetooth ECG. RDTs were read locally while ECGs were sent to a cardiology clinic which transmitted reports within 24 h from recording. Results: Among 140 people (54 men, median age 38(interquartile range 23–54) years), 98 (70%) were positive for *Trypanosoma cruzi* infection, with a linear, age-dependent, increasing trend (*p* < 0.001). Twenty-five (18%) individuals showed ECG abnormalities compatible with CD. Prevalence of ECG abnormalities was higher in infected individuals and was associated with higher systolic blood pressure and smoking. Following screening, 22 (16%) individuals underwent clinical evaluation and chest X-ray and two were referred for further evaluation. At multivariate analysis, positive CSP results (OR = 4.75, 95%CI 1.08–20.96, *p* = 0.039) and smoking (OR = 4.20, 95%CI 1.18–14.92, *p* = 0.027) were independent predictors of ECG abnormalities. Overall cost for screening implementation was <10 $. Conclusions: Combined mobile-Health and RDTs was a reliable and effective low-cost strategy to identify patients at high risk of disease needing cardiologic assessment suggesting potential future applications.

## 1. Introduction

Chagas disease (CD), caused by infection with the protozoan parasite *Trypanosoma cruzi*, is the neglected tropical disease exerting the highest burden in most Latin American countries, with 8 million persons chronically infected and approximately 200,000 new cases each year [1]. It is transmitted to humans through the feces of infected hematophagous triatomine insects in areas in which the disease is endemic and, occasionally, by non-vectorial mechanisms such as blood transfusion, organ transplants, or vertically from mother-to-child [2].

Three clinical stages of CD have been described: the acute phase, typically asymptomatic and short-lasting, followed by a chronic long-acting phase that may span for decades without showing any symptoms associated to the infection (indeterminate stage), and the determinate phase. Approximately 40% of chronically infected individuals progress to either advanced cardiac and/or digestive tract forms characterized by high morbidity and mortality, if left untreated [2].

Despite progress in vector control [3,4], a timely and accurate diagnosis remains a major obstacle to start treatment. Still today, early accessing to presently available drugs is a major issue. It is estimated that current chemotherapies only reach 1% of infected individuals [1,5].

Communities with intense transmission remain, especially in the Bolivian Gran Chaco (estimated infection rate at 4% per year) [3,4]. Cardiac involvement, i.e., Chagas Cardiomyopathy (CChC), is the main cause of death [2,5,6]. The early signs of Chagas cardiomyopathy are typically conduction system abnormalities, most commonly right bundle branch block (RBBB), often progressing to bifascicular blocks. Later manifestations include left ventricular systolic dysfunction, apical aneurysms, high-degree atrioventricular block, and sustained and non-sustained ventricular tachycardia [6,7,8]. Of note, sudden cardiac death may occur at any moment, including early phases. Therefore, early recognition of cardiac involvement through cost-effective screening efforts becomes a priority in areas with high endemic burden. As patients with CD, compared to non-CD subjects, have almost a threefold higher prevalence of electrocardiogram (ECG) alterations, ECG coupled with a rapid diagnostic test (RDT) screening can be a reasonable first-line approach. However, limited resources, lack of trained personnel and infrastructures in highly endemic areas, challenge the implementation of such programs. Smartphone technology, with its’ computational power, applied to telemedicine may overcome several of these limitations by providing an easy and affordable access to accurate diagnostic methods [9,10,11].

The aim of this study was to understand the potential impact and sustainability of a mHealth ECG screening program, coupled with an RDT test, in remote rural villages of the Bolivian Chaco with the help of a validated smartphone-based ECG (D-Heart^®^). The present device allows low-cost ECG screening campaigns by community health workers and offers the possibility of Remote ECG interpretation by expert physicians.

## 2. Materials and Methods

### 2.1. Study Population and Settings

The study was carried out in Palmarito Community (municipality of Gutierrez, Santa Cruz Department; 19°49′ S; 63°48′ W, Bolivian Chaco Region), in February 2019. In this region, estimated seroprevalence of Chagas Disease is 50% in the general population, but can be as high as 70% in individuals aged >15 years [3,4]. The nearest secondary level Hospital is located 80 km far away (Hospital Municipal de Camiri). All individuals ≥ 15 years old were invited to participate to the study. Overall, 653 inhabitants live in Palmarito, of whom 402 people ≥ 15 years old. A representative sample of 140 healthy volunteers were consecutively enrolled, taking into account the age group distribution. Demographic data was recorded, and a brief clinical history, focused on common cardiovascular risk factors and manifestations, was obtained through a standardized questionnaire. Height and weight were recorded and body mass index (BMI) calculated. All participants underwent blood pressure (BP) measurement, by trained personnel, before performing electrocardiographic and serological screening; those with elevated systolic (SBP ≥ 140 mmHg) and/or diastolic blood pressure (DBP ≥ 90 mmHg) had a second measurement.

### 2.2. ECG Screening and Referral Path

For each participant, an ECG was recorded using D-Heart^®^ electrocardiograph. D-Heart^®^ is a CE marked multiple lead smartphone-based ECG device (DI, DII, DIII, aVR, aVL, aVF peripheral leads V2 and V5 precordial leads) specifically designed for ECG screening in low-income settings by non-medical personnel [10,11]. The device is manufactured by the social-vocation start-up D-Heart, an Italian-based company. The device weighs less than 194 g and is extremely portable. If operated by non-health professionals it can register an 8-lead ECG, whereas in the health professionals setting standard 12-lead ECG can be acquired. The module Bluetooth Low Energy streams the ECG data to the smartphone in a medically certified App that enables in loco reading of the tracings or Telecardiology Reporting via web-based Telecardiology Platform. The actual components of the device offer a manufacturing price of 90$ per unit.

ECG tracings were acquired with D-Heart Smartphone ECG device during the on-site screening activities and were sent daily to the Cardiomyopathy Unit, Careggi Hospital, Florence, Italy where they were read with D-Heart Telecardiology Platform within 24 h by two staff physicians, N.M and C.F., blinded for subjects’ *T.cruzi* infection status. Each ECG was recorded with a dedicated smartphone protected by a code known by the Community Health Worker. An abnormal ECG suggestive of CChC was defined as an ECG with (i) ventricular conduction defects: complete right BBB (RBBB), left anterior fascicular block, left posterior fascicular block, left bundle branch block, or bifascicular block; (ii) any degree of atrioventricular block; (iii) rhythm disturbances: atrial fibrillation/flutter, junctional rhythm, sinus bradycardia with heart rate < 50 beats/min, or complex ventricular ectopy; (iv) other: pathologic Q waves, fragmented QRS, low QRS voltage [6,7,12]. Other findings, such as incomplete RBBB, atrial ectopy, nonspecific ST-T wave changes, right or left ventricular hypertrophy were considered nonspecific for CChC and were not included in our definition of CChC-related ECG abnormality. Reports were sent back daily to the Community Health Center in Palmarito.

### 2.3. T. cruzi Infection Screening Chagas Stat-Pak^®^ Assay

After ECG testing, all patients during the onsite screening activities performed a Chagas Stat-Pak^®^ (CSP) (Chembio Diagnostic System, Medford, NY, USA), an immunochromatographic, qualitative, rapid diagnostic test (RDT), which uses a combination of antigens for the detection of IgG antibodies to *T. cruzi*, in use as standard tool for Chagas disease screening by the Chagas National Program since 2005. Blood samples were obtained by finger-prick and the result read after 15 min, according to the manufacturer’s instructions. During previous studies, carried out in the same highly endemic area of the Bolivian Chaco, CSP yielded excellent performance in comparison with the conventional serology, with sensitivity, specificity, positive predictive value and negative predictive value up to 100%, 99.3%, 99.5%, and 100%, respectively [13,14].

### 2.4. Sustainability and Cost of a mHealth Screening Campaign

Financial feasibility models were built to project the overall cost of our screening campaign. A total of two analyses were performed: the first model would include start-up and operative costs related to human resources, consumables (RDT kits, electrodes, disinfection kits), non-consumable devices (D-Heart^®^, compatible smartphone, blood pressure cuff, and internet connection) and logistics; the second one would comprise only consumables and human resources for screening continuation.

### 2.5. Statistical Analysis

Statistical analysis of the data was performed with STATA 11.0 (StataCorp, College Statio, TX, USA). Frequencies and percentages with 95% confidence intervals (CI) for categorical variables, means and medians and interquantile ranges (IQR) for continuous variables were calculated. T-Student test or Mann–Whitney test were used to compare continuous variables. Chi-square test, or Fisher’s exact test, when appropriate, were used to investigate the association between positive CSP test with ECG abnormalities, individual risk factors and demographic data. Multivariate logistic regression was performed including age, sex and all the variables significantly associated to ECG abnormalities at univariate analysis. Results were considered significant when the *p*-value ≤ 0.05.

### 2.6. Ethics Statement

The study was realized in agreement with the Ministry of Health of the Plurinational State of Bolivia (Convenio Ministerio de Salud y Deportes, Estado Plurinacional de Bolivia/Cátedra de Enfermedades Infecciosas, Universidad de Florencia, Italia), the Servicio Departamental de Salud (SEDES) of Santa Cruz and with the support of the Guaraní political organization (Asamblea del Pueblo Guaraní). The study was approved by a local Ethic Committee and a written informed consent was obtained by each enrolled participant (or by a parent or a legal guardian, if minor).

## 3. Results

### 3.1. Baseline Characteristics

Of the 140 subjects included in the study, 54 (39%) were men, with a median age of 38 (interquanrtile range 23–54) from 15 to 85 years old. Twenty-four (17%) had family history for cardiovascular diseases and 11 (8%) of sudden unexpected death. Cardiovascular risk profile was generally low, with only five (4%) individuals affected by Type 2 diabetes mellitus, two (1%) with known dyslipidemia and median BMI was 24 kg/m2 (22–27). Palpitations were reported by 39 (28%) patients, whereas chest pain was the most common complaint, present in 82 (59%) patients. History of loss of consciousness was present in 14% of patients (Table 1)

No one had been screened with an ECG or for *T. cruzi* before the study enrolment.

### 3.2. Outcome of Combined T. cruzi and ECG Screening

Community screening was carried out in 6 days. RDTs were read locally, and results recorded, while ECGs were sent to the Florence Cardiomyopathy Unit and analyzed within 24 h (average response time: 9 ± 1 h). No ECG recording was lost, and all patients with positive ECG and RDT results combined were actively referred to further evaluation.

Overall, 98 (70%) subjects were screened positive for *T.cruzi* infection with CSP (Table 2). *T. cruzi* seroreactive people were significantly older than uninfected ones (*p* < 0.001), with a linear, age-dependent, increasing trend (*p* < 0.001). Ten subjects (7%; 51 to 85 years) had elevated BP values: four had arterial hypertension at two successive measures, and six had isolated systolic hypertension (SBP ≥ 140 mmHg, DBP < 90 mmHg). Of note, only two were aware of their condition. Mean SBP and BDP values were slightly—but significantly—higher in CSP-positive group than in non-reactive people (Table 2).

None of the study participants had performed an ECG test prior to enrolment. A total of 115 (82%) subjects had a normal ECG, while 25/140 (18%) showed ECG abnormalities, compatible with CD, and prevalence was higher in CSP positive individuals (22% vs. 7%, *p* = 0.03). No differences were described for PR and QTc intervals duration. ECG abnormalities included Bundle Branch Blocks (*n* = 8), 1st Degree Atrioventricular blocks (*n* = 3), rhythm disturbances (*n* = 5), pathologic Q waves (*n* = 2), fragmented QRS (*n* = 5), and low QRS voltage (*n* = 2) (Table 2). ECG abnormalities were directly associated with higher systolic blood pressure (117 ± 13 vs 110 ± 15, *p* = 0.017) and smoking habit (48 vs 26%, *p* = 0.030) (Table 3). Age- and sex-adjusted multivariate analysis confirmed the association of ECG abnormalities with CSP-positive results (OR: 4.75, 95%CI 1.08–20.96, *p* = 0.039) and smoking habit (OR: 4.20, 95%CI 1.18–14.92, *p* = 0.027).

### 3.3. Medical Referral, Feasibility of Current Screening Strategy, and Cost Analysis

**Figure 1:**
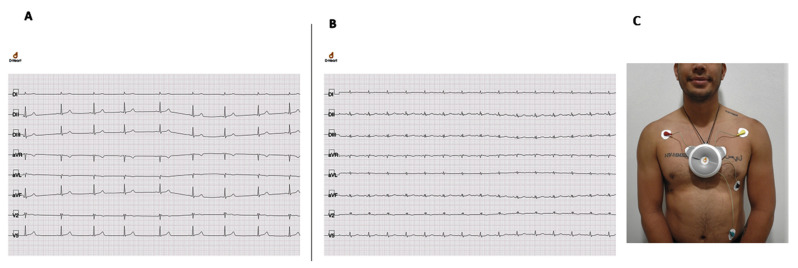
ECG samples of patients referred to further second level examinations and Smartphone ECG device used in the screening activities. (**A**) 45-year old man, active smoker with history of chest pain; his ECG showed sinus bradycardia with a RBBB; (**B**) 59-year old woman, with history of palpitations, leg oedema, chest pain and loss of consciousness; at ECG, a RBBB and low voltages were present. (**C**) Patient during screening activities with D-Heart Smartphone ECG device operating. The D-Heart device can record either 8- or 12-lead ECGs. In the 8-lead setting, DI, DII, and DIII leads are directly measured, as well as precordial unipolar leads V2 and V5. Augmented leads aVR, aVL, and aVF are calculated by definition. In the 12-lead settings, the unipolar electrode in V5 is sequentially placed in V6, V4, V3, V1 position to acquire all the precordial recordings.

Twenty-two patients with a positive CSP testing and possible CD-related ECG abnormalities were recalled from Palmarito Community and referred to the second level Camiri Hospital, where physical examination and chest X-ray were performed. All 22 patients had CD diagnosis confirmed by Chagatest Lisado ELISA (Wiener Laboratories, Rosario, Argentina), performed at the “Elvira Wunderlich” Health Center, Santa Cruz, Bolivia. Of these, two patients had cardiomegaly on the chest X-ray and were referred to further third level examinations. The first person was a 45-year old man, active smoker with history of chest pain; his ECG showed sinus bradycardia with a RBBB. The second person was a 59-year old woman, with history of palpitations, leg edema, chest pain, and loss of consciousness; at ECG, a RBBB and low voltages were present (Figure 1A–C). People with positive CSP, but normal ECG findings, were referred to the Chagas National Program for serological confirmation and possible benznidazole treatment, and managed according to their guidelines [15].

Two models for cost effectiveness analysis were developed. The first one, comprising start-up and operative costs, is summarized in Table 4. For a 6-day screening for a community of 150 inhabitants, the overall start-up amount was projected to 4.82$/patient and to 8.23$/patient when operative costs (i.e., on-site nurse and healthcare assistant with remote physician on call) were included. For the second model, intended to predict cost of screening continuation, an average of 5.13$/patient was estimated.

## 4. Discussion

In this study, we evaluated the feasibility of a combined mobile-health electrocardiographic and rapid diagnostic test screening for Chagas-related cardiac alterations in a in a low-income setting, hyperendemic for CD. Subjects screened with ECG were also tested for the presence of *T.cruzi* antibodies, by an easy-to-use RDT.

In the surveyed community, seroprevalence for *T. cruzi* was 70%, and its distribution by age-class was consistent with previously reported data from this [3,4].

More than one in five patients with CSP positive serology showed ECG abnormalities compatible with CChC (*n* = 22/98, 22%), in line with the estimate that 20–30% of infected individuals eventually develop heart disease. The most common findings were ventricular conduction defects, including RBBB and left anterior fascicular block. Moreover, we observed a number of ECG with fragmented QRS, considered as a predictor of arrhythmic events in patients with ischemic and non-ischemic cardiomyopathy, previously reported to be highly prevalent among patients with advanced CChC [6,7]. Other abnormalities included AVB and rhythmic disturbances, which are typical CChC manifestations, and low QRS voltage, which has been previously identified as a strong predictor of the risk of death from cardiac causes in CD patients [12].

Notably, 2 of the 22 individuals with positive ECG and CSP were referred for further medical evaluation: in both cases, ECG showed at least two alterations and chest X-ray was abnormal. Multiple ECG abnormalities have already been described as highly prevalent in patients with signs of dilated cardiomyopathy at echocardiogram [12].

Overall, our observations strongly emphasize the potential application of mHealth technology and telemedicine, together with RDT, to improve access to diagnosis and treatment for CD and CChC in remote areas of the rural Bolivian Chaco. In fact, although a pilot study, simultaneous screening by CSP and D-Heart electrocardiograph resulted feasible, with a cost/patient < 10$ to start up.

The combined, on-field use of RDT and ECG in large-scale screening campaigns could play a pivotal role within a more comprehensive strategy against CD. Early diagnosis of CD is of paramount importance to start treatment before symptoms progress. In remote regions, easy-to-use RDTs, which use whole blood from digital puncture as sample, would ease access to CD diagnosis, allowing timely treatment.

Recently, the use of combined RDTs was shown to be a reliable and accurate alternative to conventional serological assays in order to achieve a conclusive CD diagnosis, in settings where equipped labs and trained personnel are not available [13].

The role of antitrypanosomal treatment in adult patients with established CChC remains controversial. So far, the only published placebo-controlled trial in adults with advanced CChC concluded that benznidazole treatment did not affect the clinical progression of Chagas cardiomyopathy, but important methodological bias has been raised [16,17]. Ideally, etiological treatment should be offered timely in adult patients with chronic Chagas disease before established cardiac damage requires more aggressive management [18,19]. Furthermore, recently published studies support benznidazole use in standard treatment in addition to new alternative regimens for short-course and combination treatments [20].

Screening campaigns that result in early therapy inception are, however, successful as long as effective vector control activities can be achieved, and intensive care be delivered to individuals in need.

As a case in point, in 2013, blanket insecticide application was shown to decrease the force of infection in the Bolivian Chaco, though active transmission remained [3,21,22]. Moreover, several pharmacological and non-pharmacological interventions are currently available and have been increasingly used in CChC patients with the intention of preventing or delaying complications [23].

**Figure 2:**
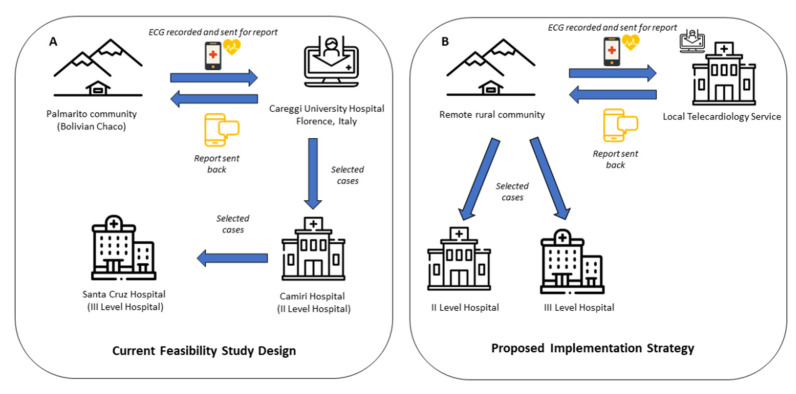
Current Study Design and Proposed on site implementation strategy. (**A**) Current study design is summarized. Specifically, patients are screened on site, in a community of the Bolivian Chaco. ECGs are sent to Telecardiology Service in Careggi University Hospital, Italy and reports are back on site; in case of need, patients with a pathologic ECG were recalled back in the community and referred to the nearest second level Hospital in Camiri (distance 80 km). Patients requiring higher level of care were further referred to a III level Hospital in Santa Cruz (distance 250 km). (**B**) Proposed implementation strategy is described. Patients are screened on site and ECGs are sent to a local telecardiology service; in case of need, patients with a pathologic ECG are recalled back in the community and referred to a second or third level Hospital, according to their condition.

As part of the study protocol, ECG recordings were sent to Florence for analysis. It is tempting to hypothesize that, should combined (ECG and RDTs) screening programs be further implemented, ECGs could be seamlessly transmitted to local cardiologists or community physicians with the intention to monitor individuals through time and create electrocardiographic and serologic ‘profiles’ to detect conversion (Figure 2A,B).

Finally, pocket echocardiography integrated mHealth device assessments are now under scrutiny for potential applications in resource-limited settings. In a randomized trial enrolling 253 patients at a tertiary care center in Bangalore, India, patients who were randomly allocated to a m-health clinic for valvular and structural heart disease, as opposed to standard of care, were associated with shorter time to definitive therapy [24].

In this scenario, adding such instruments to CD screening would allow to reduce lag from infection to diagnosis, increase access to therapy and improve outcomes in patients with signs compatible with early cardiomyopathy, thus limiting disease progression and morbidity.

Ultimately, our effort may focus on bringing high-tech instruments at low-cost for effective remote screening therefore allowing for appropriate and timely diagnosis.

The study has limitations. The healthy volunteers were not randomly selected, but consecutively enrolled in the Health Centre. Severely ill community residents may have been unable to report to the health Centre for evaluation. Moreover, screening for CD was made on a single RDT, namely, CSP assay, which is in use as standard tool for Chagas disease screening by the Chagas National Program since 2005 and showed an excellent performance in the same geographical area [13,14]. Only people with potential CD-related ECG abnormalities (*n* = 22) were referred to a secondary level hospital for further investigations, including serology confirmation by ELISA testing. People with positive CSP, but normal ECG findings, were referred to the Chagas National Program for serological confirmation and were offered benznidazole treatment, but such data were not collected, being beyond the objective of the study.

## 5. Conclusions

Early diagnosis of CD and CChC is of paramount importance to provide access to targeted therapy (currently <1% of all seropositive subjects) and maximize treatment benefits. Combined mHealth and RDTs may prove reliable and effective low-cost strategies, especially in rural, highly endemic environments like the Bolivian Chaco, to identify patients at high risk of disease and in need of further cardiologic assessment. Further studies are clearly needed to assess if these theoretical advantages are supported by patient-centered outcomes and positive cost–benefit analysis.

## Figures and Tables

**Table 1:** Characteristics of the surveyed population in a rural community of the Bolivian Chaco by *T. cruzi* infection status.

	Total (*n* = 140)	Chagas Stat Pak^®^		*p*-Values
		Negative (*n* = 42)	Positive (*n* = 98)	
Sex				
Male	54 (39%)	22 (41%)	32 (59%)	
Female	86 (61%)	20 (23%)	66 (77%)	0.028 *
Age	38.5 (23–54)	20 (17–30)	45 (33–62)	<0.001 ^#^
Range	15–85	15–75	15–85	
15–20 yo	34	23 (68%)	11 (32%)	
21–49 yo	63	17 (27%)	46 (73%)	
≥50 yo	43	2 (5%)	41 (95%)	<0.001 ^§^
SBP (mmHg)	111 ± 16	110 ± 15	117 ± 13	0.010 °
DBP (mmHg)	69 ± 10	67 ± 8	70 ± 10	0.048 °

Legend: IQR: interquartile range; yo: year-old; SBP: systolic blood pressure; DBP: diastolic blood pressure; * Pearson’s chi-squared test; ^#^ Mann–Whitney test; ^§^ linear regression; **°** *t*-Student test.

**Table 2:** Electrocardiographic findings in *T. cruzi* infected and uninfected population of a rural community of the Bolivian Chaco.

	Total (*n* = 140)	Chagas Stat Pak^®^	
		Negative (*n* = 42)	Positive (*n* = 98)
Normal	115 (82%)	39 (93%)	76 (78%)
Any CD-related abnormality	25 (18%)	3 (7%)	22 (22%)
Bundle Branch Blocks			
Complete right BBB	6 (4%)	0	6 (6%)
Left anterior fascicular block	2 (1%)	0	2 (2%)
Atrioventricular Blocks			
I degree atrioventricular block	3 (2%)	1 (2%)	2 (2%)
Rhythm disturbances			
Sinus bradycardia	3 (2%)	1 (2%)	2 (2%)
Complex ventricular ectopies	2 (1%)	0	2 (2%)
Other			
Pathologic Q waves	2 (1%)	0	2 (2%)
Fragmented QRS	5 (4%)	1 (2%)	4 (4%)
Low QRS voltage	2 (1%)	0	2 (2%)
Heart rate (bpm) (±SD)	70 ± 10	73 ± 12	69 ± 9
PR interval (ms) (±SD)	156 ± 21	156 ± 16	159 ± 22
QTc interval (ms) (±SD)	410 ± 24	408 ± 25	411 ± 23

Legend: CD: Chagas disease; BBB: bundle branch blocks; AVB: atrioventricular block; bpm: beats per minute; ms: milliseconds; SD: standard deviation.

**Table 3:** Characteristics and cardiovascular risk factors of the surveyed population in a rural community of the Bolivian Chaco, by D-Heart^®^ result.

	Total (*n* = 140)	D-Heart^®^ ECG		*p*-Value
		Normal (*n* = 115)	Any Abnormality (*n* = 25)	
Sex				
Male	54 (39%)	44 (38%)	10 (40%)	0.871 *
Age				
Median (IQR)	38 (22–54)	37 (20–53)	40 (31–59)	0.92 ^#^
Range	15–85	15–85	16–83	
Chagas Stat Pak^®^				
Negative	42	39 (34%)	3 (12%)	
Positive	98	76 (66%)	22 (88%)	0.032 ^^^
CV risk factors				
Family history of CV diseases	24 (17%)	22 (19%)	2 (8%)	0.247 ^^^
Family history of sudden death	11 (8%)	11 (10%)	0	0.211 ^^^
Positive Smoking History	42 (30%)	30 (26%)	12 (48%)	0.030 *
Dyslipidaemia	2 (1%)	1 (0.9%)	1 (4%)	0.326 ^^^
Diabetes	5 (4%)	4 (3.5%)	1 (4%)	0.632 ^^^
History of leg oedema	54 (39%)	41 (36%)	13 (52%)	0.128 *
Loss of consciousness	20 (14%)	16 (14%)	4 (16%)	0.757 ^^^
History of Palpitations	39 (28%)	33 (29%)	6 (24%)	0.635 *
History of Chest pain	82 (59%)	66 (57.4%)	16 (64%)	0.543 *
BMI (IQR)	24 (22–27)	24 (22–27)	24 (22–28)	0.389 ^#^
SBP (mmHg)	111 ± 16	110 ± 15	117 ± 13	0.017 °

Legend: CV: Cardiovascular Disease; IQR: Interquartile range; BMI: Body Mass Index; SBP: Systolic Blood Pressure. * Pearson’s chi-squared test; ^#^ Mann–Whitney test; ^^^ Fisher’s exact test; ° *t*-Student test.

**Table 4:** RDT and ECG screening model for 150 Individuals for a 6-day screening.

Start Up Cost				
	Item	Cost/Unit	Units Needed	Total Cost ($)
Consumables	D-Heart	280	1	280
	Smartphone	200	1	200
	Electrodes	0.12	150	18
	Blood Pressure Cuff	20	1	20
	Rdt	1	150	150
	Disinfection Kit	2	5	10
	Data Plan (Sim Card) 10 GB for 30 Days	40	1	40
Operative Costs	Nurse	100	1	100
	Healthcare Assistant	60	1	60
	Remote Physician	330	1	330
	Transportation (200 Km)	11	6	66

## Data Availability

The data presented in this study are available on request from the corresponding author.
